# Novel considerations on EGFR-based therapy as a contributor to cancer cell death in NSCLC

**DOI:** 10.3389/fonc.2023.1120278

**Published:** 2023-02-23

**Authors:** Weiwei Peng, Chengyun Yao, Qin Pan, Zhi Zhang, Jinjun Ye, Bo Shen, Guoren Zhou, Ying Fang

**Affiliations:** ^1^ Department of Medical Oncology, Jiangsu Cancer Hospital & Jiangsu Institute of Cancer Research & The Affiliated Cancer Hospital of Nanjing Medical University, Nanjing, China; ^2^ Department of Radiation Oncology, Jiangsu Cancer Hospital & Jiangsu Institute of Cancer Research & The Affiliated Cancer Hospital of Nanjing Medical University, Nanjing, China; ^3^ Department of Medical Oncology, Liyang People’s Hospital, Liyang, China; ^4^ Department of Thoracic Surgery, Jiangsu Cancer Hospital & Jiangsu Institute of Cancer Research & The Affiliated Cancer Hospital of Nanjing Medical University, Nanjing, China

**Keywords:** non-small cell lung cancer (NSCLC), non-coding RNAs, epidermal growth factor receptor (EGFR), cell death, combined treatment

## Abstract

Epidermal growth factor receptor (EGFR)-tyrosine kinase inhibitors (TKIs) represented by gefitinib and erlotinib are widely used in treating non-small cell lung cancer (NSCLC). However, acquired resistance to EGFR-TKI treatment remains a clinical challenge. In recent years, emerging research investigated in EGFR-TKI-based combination therapy regimens, and remarkable achievements have been reported. This article focuses on EGFR-TKI-based regimens, reviews the standard and novel application of EGFR targets, and summarizes the mechanisms of EGFR-TKI combinations including chemotherapy, anti-vascular endothelial growth factor monoclonal antibodies, and immunotherapy in the treatment of NSCLC. Additionally, we summarize clinical trials of EGFR-TKI-based combination therapy expanding indications to *EGFR* mutation-negative lung malignancies. Moreover, novel strategies are under research to explore new drugs with good biocompatibility. Nanoparticles encapsulating non-coding RNA and chemotherapy of new dosage forms drawn great attention and showed promising prospects in effective delivery and stable release. Overall, as the development of resistance to EGFR-TKIs treatment is inevitable in most of the cases, further research is needed to clarify the underlying mechanism of the resistance, and to evaluate and establish EGFR-TKI combination therapies to diversify the treatment landscape for NSCLC.

## Introduction

Lung cancer is the leading cause of cancer-related deaths worldwide. According to global cancer statistics, approximately 350 deaths occurs every day from lung cancer in 2022 (1). The pathogenesis of lung cancer is very complex, involving many factors, such as environmental factors, genetic factors, and lifestyle factors such as smoking. According to histological classification, lung cancer can generally be divided into two subtypes: small cell lung cancer and non-small cell lung cancer (NSCLC). About 15% of lung cancer cases are small cell lung cancer, while NSCLC accounts for about 85% of lung cancer cases and the majority of lung cancer morbidity and mortality (2). NSCLC can be further divided into adenocarcinoma, squamous cell carcinoma, and large cell carcinoma, among which adenocarcinoma is the most common, accounting for about 40% of all NSCLC cases (3). For patients who are diagnosed with early-stage NSCLC, the survival rate is relatively high after surgery (4). However, in many cases, patients with early-stage NSCLC do not show typical clinical symptoms, making early screening of NSCLC very difficult, resulting in more than half of NSCLC patients already developed local or distant metastases at diagnosis (5). At present, the main treatment strategy for NSCLC is still based on surgical resection, combined with adjuvant chemotherapy, radiotherapy, immunotherapy, and targeted therapy. However, only about 20-30% of patients are clinically suitable for surgical treatment (6), and the recurrence rate after surgery is relatively high. In addition, conventional chemotherapy-based regimens are associated with limited efficacies in clinical practice, and radiotherapy is associated with side effects and reduced quality of life. Due to these limitations of conventional treatment strategies, the development of novel treatment for NSCLC represents a pressing unmet need. With the development of molecular biology, cancer-targeted therapy in NSCLC has gained increased attention. Considering the significant heterogeneity of NSCLC, treatment regimens combining targeted therapies and other conventional or novel treatment strategies might serve to improve the clinical outcomes of patients with NSCLC (7).

**Figure 1:**
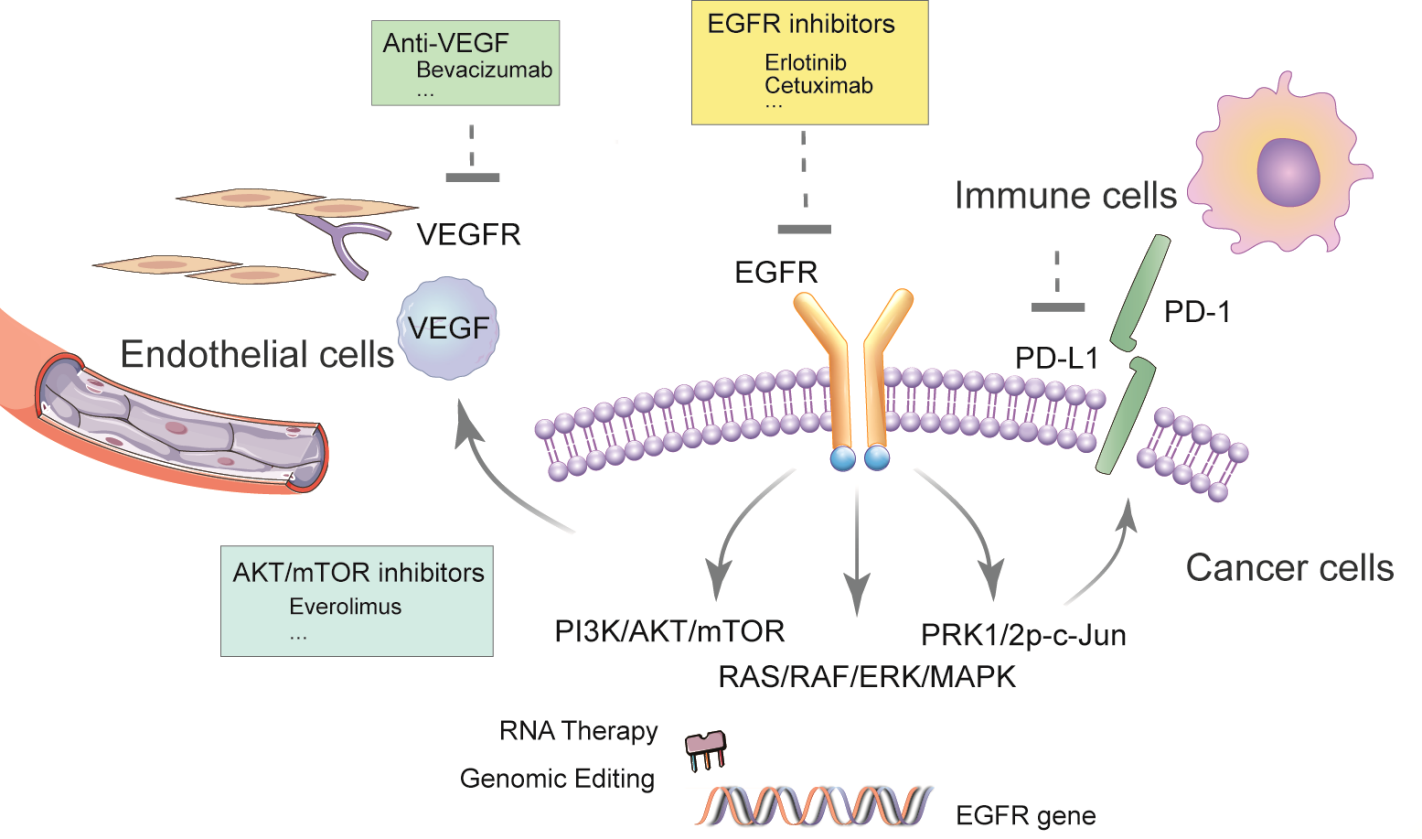
Schematic of EGFR-TKI-based regimen in NSCLC.

The emergence of epidermal growth factor receptor (EGFR)-tyrosine kinase inhibitors (TKIs) represented by gefitinib provides a new option for the treatment of advanced NSCLC. However, the response rate of EGFR-TKI in advanced NSCLC remains moderate. In the FLAURA trial, the progression-free survival (PFS) was only 10.2 months in patients with *EGFR* mutation-positive (exon 19 deletion or L858R) advanced NSCLC who received standard EGFR-TKI as first-line treatment (8). In another Phase 3 study, the PFS of gefitinib alone in newly diagnosed metastatic NSCLC with *EGFR* mutation was only 11.9 months with the objective response rate (ORR) being 67%, while in the combination group where patients were treated with gefitinib combined with carboplatin plus pemetrexed, ORR was 84% and PFS nearly doubled (20.9 months) (9). Patients treated with gefitinib will inevitably develop resistance, making it an unsustainable regimen (10). Moreover, rebiopsy of tumor tissue demonstrated that EGFR-TKI administration was related to changes in the tumor microenvironment, resulting in a reduced proportion of anti-tumoral CD8+ and FOSP3+ tumor-infiltrating lymphocytes, increased PD-L1 expression, and tumor mutation burden (11). This report reviews the prospects of subsequent immunotherapy after EGFR-TKI treatment, and the necessity of the combined application of EGFR-TKI and other drugs, which not only gives full play to the respective advantages of drugs but also presents potential synergistic effects against refractory malignancies. Therefore, the combination therapy has achieved good effects and has attracted much attention (See **Figure 1**).

## EGFR-TKI overview

EGFR is the protein encoded by the proto-oncogene c-erbB1, which is a member of the EGFR receptor family and is highly expressed in lung cancer. The tyrosine kinase is an essential subunit of EGFR. The transmembrane protein EGFR consists of three parts, namely the extracellular domain containing ligand binding sites, the single-transmembrane hydrophobic α-helix region, and the receptor tyrosine kinase (RTK) active intracellular domain (12–14). In the inactivated/dormant state, EGFR exists as a monomer on the cell membrane, RTK exists as a monomer and is inactive. Once the signal molecule binds to the extracellular domain of the receptor, the two form a dimer on the membrane, making the tails of the intracellular domain contact each other, thereby activating protein kinases and phosphorylating them to form signaling complexes. The extracellular region of EGFR binds to extracellular ligands to form dimers of EGFR monomers and other EGFR molecules or other ErbB family protein receptors. After dimerization, the intracellular tyrosine kinase region of EGFR binds to a molecule of ATP, which activates this region after phosphorylation. Subsequently, specific tyrosine residues on the C-terminus are phosphorylated through autophosphorylation or transphosphorylation. These phosphorylated sites provide binding sites for other downstream signaling molecules and activate the downstream signaling pathways RAS-RAF-MEK-ERK and RAS-PI3K-PTEN-AKT-mTOR, thereby promoting tumor cell proliferation, differentiation, and migration (15–18). This signal complex activates a series of intracellular biochemical reactions *via* RAS/RAF/ERK/MAPK pathway and PI3K/PKB/AKT pathway, triggering transcription of genes regulating the growth and differentiation of target cells (19).

The first generation EGFR-TKIs, represented by gefitinib and erlotinib, can compete for the adenosine triphosphate (ATP) binding site on the catalytic region of EGFR-TKI, inhibit RTK activity, and block the phosphorylation of RTK itself and its substrates. In turn, it blocks the conduction of downstream RAS/RAF/ERK/MAPK signaling pathways and inhibits the growth, proliferation and differentiation of tumor cells (20). The efficacy of erlotinib is slightly better than that of gefitinib, especially in patients with brain metastases from lung cancer, as a result of smaller molecular weight and better ability to cross the blood-brain barrier (21). Second-generation EGFR-TKIs Afatinib (BIBW2992) and dacomitinib (PF00299804) are oral ErbB family blockers that inhibit EGFR signaling by irreversibly binding to the ATP domain of the ErbB family kinase domain access (22, 23). A Phase 3 clinical trial demonstrated superior efficacy of dacomitinib as the first-line treatment for patients with *EGFR* mutation-positive NSCLC: the median PFS was 14.7 months in the dacomitinib group and 9.2 months in the gefitinib group, suggesting a better therapeutic efficacy as a second-generation EGFR-TKI (24). The third-generation EGFR-TKIs, represented by osimertinib, mainly form a covalent bond through an unsaturated acryl chain, targeting the cysteine-797 residue of the ATP binding site, and irreversibly interact with the catalytic active center of the EGFR receptor. The covalent binding of EGFR inhibits both the phosphorylation of EGFR kinase and the phosphorylation of downstream signaling substrates AKT and ERK, selectively and irreversibly targeting EGFR-sensitive mutations and T790M resistance mutations in patients with NSCLC. Osimertinib yielded promising clinical outcomes among patients with NSCLC harboring *EGFR* T790M mutation (25).

Studies have shown that EGFR-TKI monotherapy is safe and effective in *EGFR* mutation-positive NSCLC (mainly in exons 19 and 21), but not as effective in *EGFR* mutation-negative NSCLC (26). For patients with *EGFR* mutation-negative NSCLC, the recommended treatment still centers on chemotherapy, and a meta-analysis showed significant increase in PFS and ORR, but not in OS. This study suggested that chemotherapy remains a better treatment option than EGFR-TKI for patients with *EGFR* mutation-negative NSCLC. Docetaxel and pemetrexed are standard second- and third-line treatment options for patients with *EGFR* mutation-negative NSCLC. Erlotinib can also be used as a second- and third-line treatment option for such patients, especially for those with poor ECOG PS scores who cannot tolerate the side effects of chemotherapy.

Small-molecule drugs targeting the RTK is a breakthrough in the treatment of NSCLC, despite that the efficacy of monotherapy is not yet convincing. Additionally, acquired drug resistance becomes prominent with prolonged treatment, greatly affecting drug efficacy. EGFR-TKI resistance includes primary resistance and secondary resistance. Patients who do not respond to EGFR-TKIs or respond for a very brief period of time (typically less than 3 months) are considered primary resistance (27). The underlying mechanism of primary resistance include lack of target dependency, molecular alterations of genes from downstream pathways, and in some cases, incomplete drug impact caused by pharmacokinetic therapy failures (28–30).

The effects of EGFR-TKIs vary among different *EGFR* mutation sites, which may contribute to primary resistance to EGFR-TKI treatment. Among them, exon 19 deletion mutation and exon 21 L858R point mutation are common sensitive mutations, while exon 20 insertion or repeat mutation is associated with EGFR-TKI resistance (31). Patients who develop resistance after achieving an initial response or stable disease are considered secondary/acquired resistance, which often occurs within a year from the initiation of first- and second- generation EGFR-TKIs, and about 19 months for third generation EGFR-TKIs in the first-line setting (8). The underlying mechanism of acquired resistance can be divided into point mutations in target genes, activation of alternative signaling pathways, and phenotypic or histological transformation. Point mutations of target genes and activation of alternative signaling pathways mainly include T90M, *HER2* amplification, *MET* amplification, *BRAF* amplification, *PIK3CA* mutation. As a receptor tyrosine kinase. although *HER2* amplification is found in only 1-3% of lung adenocarcinomas, it is the second common mechanism of acquired resistance to EGFR-TKIs, occurring in 12% of cases (32, 33). The *MET* gene encodes c-met, which is a receptor for hepatocyte growth factor (HGF), and the binding of HGF with c-met activates the phosphorylation of HER3 (a member of the EGFR family) and the subsequent phosphorylation of the PI3K-AKT signaling pathway, one of the downstream effector of EGFR activation (30, 34). Thus, *MET* amplification causes acquired resistance to EGFR-TKIs and promotes the development of NSCLC. Inhibition of one signaling pathway may activate alternative signaling pathways, leading to mutual substitution between signal transduction pathways, resulting in the acquisition of resistance to EGFR-TKIs. There is mutual interference between EGFR and IGF1R due to EGFR and insulin-like growth factor 1 receptor (IGF1R)-mediated activation of downstream MAPK and AKT signaling. Inhibition of EGFR subsequently activates the IGF1R alternative signaling pathway, leading acquired resistance to afatinib as well (35, 36). Collectively, though EGFR-TKIs demonstrate promising therapeutic effects, considering drug-resistant mutations and activation of alternative signaling pathways, combination therapies are greatly necessary to provides resolutions for drug resistance and new insights into the treatment of NSCLC.

## EGFR-TKI combination therapy

### Combined application of EGFR-TKI and chemotherapy

Chemotherapy can be an effective way to treat in many types of advanced malignant tumors. However, the adverse events associated with chemotherapy can be significant, resulting in many patients unable to tolerate high-dose chemotherapy. Molecular targeted therapy is to act on the identified carcinogenic sites at the cellular molecular level to so that the tumor cells can be eliminated without affecting adjacent normal cells (37). The combination of chemotherapy and targeted therapy can improve safety by reducing the dose of chemotherapy drugs, and obtain better efficacy. Gefitinib combined with pemetrexed in the treatment of human NSCLC cell lines showed antagonism in gefitinib-sensitive cell lines. However, synergistic effects of the combination therapy were observed in the gefitinib-resistant PC9/GR human NSCLC cell line, and a significant decrease in the phosphorylation level of AKT was observed, demonstrating a significantly enhanced anti-proliferative effect (38). A randomized phase II trial conducted by Aerts et al. found that compared with erlotinib monotherapy, the combination of erlotinib and pemetrexed did not significantly improve PFS in patients with recurrent NSCLC non-squamous cell carcinoma. While OS was significantly improved, this therapy is not suitable for the combination of erlotinib and decitabine in the treatment of patients with recurrent NSCLC squamous cell carcinoma (39).

The combination of EGFR-TKIs and chemotherapy targets both EGFR-TKI sensitive and resistant subgroups, respectively. EGFR-TKI in combination of chemotherapy demonstrated excellent efficacy in an *in vitro* EGFR-TKI-resistant NSCLC model. Moreover, researchers investigated the effects of drug sequence on therapeutic effects, and found that sequential chemotherapy and subsequent EGFR-TKI yielded better therapeutic effect. When EGFR-TKI and chemotherapy were given sequentially, more cytotoxicity was observed in the treatment regimen where chemotherapy was given before TKI (40). When the chemotherapy drugs are given after erlotinib, the combination demonstrated a synergistic effect, but such effect was not observed when erlotinib and chemotherapy drugs were used simultaneously. These results show that the sequential EGFR-TKI treatment of NSCLC with chemotherapy drugs is suitable for patients with EGFR-TKI-resistant NSCLC non-squamous cell carcinoma (41). Another phase 3 clinical trial showed that the median PFS was significantly longer in patients with T790M-negative and drug-resistant NSCLC who received gefitinib plus chemotherapy, when compared with chemotherapy alone (42). Furthermore, for patients with *EGFR* mutation-positive lung adenocarcinoma who developed acquired resistance to erlotinib or gefitinib, drug efficacy to the original TKI can be recovered when combining with conventional chemotherapy (43). A retrospective study of erlotinib combined with chemotherapy or chemotherapy alone showed that the RR of the two groups were 41% and 18%, respectively, but there was no significant difference in OS and PFS. Another retrospective study including 78 patients with NSCLC and acquired resistance against targeted therapy showed that the ORRs in patients who received chemotherapy alone was 18%, and 41% in patients who received erlotinib plus chemotherapy (P=0.008). In another case-control study, patients who received TKI plus chemotherapy had significantly improved OS (14.5 months *vs*. 2.0 months) compared with those who censored TKI treatment after TKI treatment failure. In addition, OS after lung cancer diagnosis also improved (54.5 months *vs*. 28.3 months).

### Combined application of EGFR-TKI and anti-vascular endothelial growth factor monoclonal antibody

Physiological angiogenesis is essential for tissue and organ to grow and maintain their function. However, the angiogenesis of malignant tumors is pathological angiogenesis, and effective inhibition of such angiogenesis can inhibit tumor growth and metastasis. Under the over-stimulation of pro-angiogenesis factors, new blood vessels are often tortuous and over-permeable, endothelial cells are loosely connected, and pericyte coverage is low, resulting in increased vascular permeability, high interstitial fluid pressure, blood perfusion and oxygenation reduction, eventually leading to hypoxia and acidity of the tumor microenvironment (44–46). The anti-tumor angiogenesis method has become a promising new strategy for tumor treatment and anti-VEGF monoclonal antibody can be used in combination with a variety of small-molecule targeted drugs and biological immune drugs (47).

A variety of vascular-related factors are related to tumor neovascularization. Under hypoxia, tumor cells can secrete a variety of pro-angiogenesis factors to promote angiogenesis. One of the most important growth factors is VEGF. VEGF can bind to receptors VEGFR-1 and VEGFR-2 on the surface of vascular endothelial cells, induce receptor dimerization, and phosphorylate the receptors’ intracellular tyrosine kinase structure domain activation, activate PLC-γ/PKC, RAS/MAPK and PI3K/AKT signaling pathways, induce endothelial cell proliferation, migration and cell survival (48–51), and ultimately lead to tumor angiogenesis (52). At the same time, VEGF is also known to play a key role in the early stages of metastases (53–55). VEGFR-2 has been recognized as the most important receptor among the family, which is the main mediator of endothelial cell function in VEGF, responsible for the development of physiological and pathological blood vessels, promoting the proliferation of cancer cells mainly through RAS/RAF/MAPK signaling pathway (56). Many signal transduction pathways affect tumor cell survival and proliferation in lung cancer. The serine/threonine-protein kinase Akt is not only an important part of the migration and survival signaling pathway, but also a strong inducer of neovascularization and endothelial cell proliferation. The Akt-mediated signaling pathway can be stimulated by a variety of angiogenic factors, and plays a key role in the survival, migration and proliferation of endothelial cells (57, 58). It can be seen that the activation of the two signaling pathways, PI3K/Akt/mTOR and RAS/RAF/MEK/Erk, plays a vital role in cell survival at all stages of cancer (59). Blocking one of these pathways is likely to make the other pathways serve as a rescue or escape mechanism for cancer cells, that is, bypassing the inhibitory effect of a single signal pathway. The activation of EGFR ligands (such as EGF or TGF-a) leads to increased expression of VEGF. Ligand is an important inducer of angiogenesis as well as EGFR-TKI resistance in *EGFR* mutation-positive lung cancer. The most likely mechanism of resistance is by enhancing PI3K/Akt signaling which coordinates with hypoxia to regulate VEGF (60). For treatment strategies aiming to overcome such resistance, only blocking one of the two approaches may not be enough to obtain meaningful results (61). Therefore, the combination of anti-VEGF monoclonal antibody and EGFR-TKI may be a valid anti-tumor strategy. Some preclinical studies have shown that the combination of EGFR and VEGFR inhibitors has an enhanced effect on lung cancer cell lines with the potential to overcome primary or acquired resistance to EGFR inhibitors (61).

Bevacizumab is a recombinant humanized anti-VEGF monoclonal antibody. Bevacizumab and erlotinib target different signaling pathways with potential complementary mechanisms that can inhibit tumor growth, and their toxicities have almost no overlap, making this combination a valid treatment strategy (62). The combination of bevacizumab and erlotinib demonstrated superior PFS in patients with *EGFR* mutation-positive stage IIIB/IV non-squamous cell carcinoma NSCLC, when compared with erlotinib monotherapy (16.0 months *vs.* 9.7 months). No significant difference in adverse reactions above grade 3 was observed in patients receiving the combination when compared with bevacizumab monotherapy, and it can be used as the first-line treatment of NSCLC with *EGFR* mutation. A study including 154 patients demonstrated that treatment of NSCLC patients with *EGFR* or *KRAS* mutations can obtain longer PFS, but OS did not improve significantly (62, 63). The phase 3 NEJ026 study from Japan has confirmed that bevacizumab combined with erlotinib can significantly prolong PFS compared with single-agent erlotinib (median PFS: 16.9 *vs.* 13.3 months). For the two most common mutations of *EGFR*, ex19del and ex21 L858R, the PFS of the combinatorial therapy was 16.6 and 17.4 months, respectively, and the PFS of erlotinib alone was 12.4 and 13.7 months (64). In addition, a national multi-center, randomized controlled, open-label phase III clinical study from China (the ARTEMIS trial [CTONG 1509]) also confirmed the efficacy of the combination therapy. The results showed that in the first-line treatment of *EGFR* mutation-positive advanced NSCLC, the median PFS in the bevacizumab plus erlotinib combination treatment group was 17.9 months, and the median PFS in the erlotinib monotherapy group was 11.2 months (65).

In summary, the combination of bevacizumab plus erlotinib is related to the state of NSCLC gene mutations and can significantly prolong PFS, but whether this improvement in PFS could translate to a longer OS requires further study. The mechanism may be that cancer cells use multiple ways to block and escape immune response (66). The reason is related to the hypoxic immunosuppressive microenvironment formed by the abnormal proliferation of tumors and blood vessels. The angiogenesis factor promotes tumor angiogenesis under hypoxia while downregulating immune response.

### EGFR-TKI combined immunotherapy

Cancer cells can establish an immunosuppressive microenvironment by inhibiting immune recognition, inducing immune cell apoptosis, and reducing the infiltration of anti-tumor immune cells (4). It can be speculated that targeted therapy induces antigens release through killing of tumor cells, which in turn initiates and enhances anti-tumor immune responses (67–69). A number of preclinical studies has suggested the potential of EGFR-TKI in combination with immunotherapy as a treatment for NSCLC. Akbay et al. have shown that the activation of the EGFR pathway can upregulate the expression of PD-L1 and other immunosuppressive factors including cytokine production and accumulation of tumor-associated macrophages, suggesting that EGFR on NSCLC cell membrane could remodel the tumor microenvironment (70). Chen et al. further explored the mechanism of this observation and suggested that EGFR activation upregulates PD-L1 through p-ERK1/2p-c-Jun, leading to the apoptosis of tumor-infiltrating T cells (71). These results indicated the possibility that in EGFR-mutated NSCLC, the immune escape in the oncogenic activation of the EGFR pathway is mainly mediated by the upregulation of PD-L1 through EGFR activation. Therefore, in NSCLC with *EGFR* mutations, EGFR-TKIs and anti-PD-1 antibodies may have similar but not synergistic effects in disrupting the PD-1/PD-L1 interaction (71). Consistent with these results, a retrospective analysis showed that the expression frequency of PD-L1 is relatively low in patients with *EGFR* mutations before EGFR-TKI exposure and acquired resistance (67). In addition, patients with *EGFR* mutations have a relatively low response rate to PD-1/PD-L1 inhibitors, and the combined expression rate of PD-L1 and CD8+ of tumor-infiltrating lymphocytes is low. The efficacy of anti-PD-L1 (topalima) in combination with chemotherapy (pemetrexed and carboplatin) in patients with *EGFR* mutations after EGFR-TKI failure was evaluated in a phase 2 clinical trial (NCT03510666). The results showed that in *EGFR* mutation-positive lung cancer, not only the density and function of CD8+ tumor-infiltrating lymphocytes are suppressed, the PD-L1 genes CD274 and CD86 are also downregulated, and HHLA2 and VTCN1 (B7-H4) were upregulated. Activation of MEK/erk pathway can upregulate B7H4, and activation of PI3K/Akt can upregulate PD-L1, suggesting that MEK/erk and PI3K/Akt signaling pathways are involved in *EGFR* mutation-positive lung cancer (72). Further research is needed to clarify these inconsistent results of the combined application of EGFR-TKI and anti-PD-1 or anti-PD-L1 in these preclinical studies.

## EGFR-TKI combination therapy clinical trial

### Combined application of EGFR-TKI and chemotherapy drugs

BIM deletion polymorphism might be associated with a poor clinical response to EGFR-TKIs in patients with *EGFR* mutation-positive NSCLC. The study NCT02859077 is designed to explore an EGFR-TKI (gefitinib) in combination with chemotherapy as first-line treatment in stage IIIB/IV NSCLC patients with both *EGFR* mutation and BIM deletion polymorphism. Another study HS-10296-306 (NCT04923906) is designed to evaluate the safety and efficacy of aumolertinib, a third generation EGFR-TKI, in combination with chemotherapy in the treatment of locally advanced or metastatic NSCLC with sensitizing EGFR mutations, when compared to aumolertinib alone (**Table 1**).

**Table 1:** Clinical trials studying EGFR-TKI-based combination regimens in the NSCLC.

Identifier	Study phase	Study design	Class of agents	Agents	Participants	Estimated enrollment	Primary endpoint	Status
**NCT02859077**	Phase 3	Single arm, open label	EGFR-TKI Chemotherapy	Gefitinib, Pemetrexed, Gemcitabine, Carboplatin	NSCLC	100	PFS	Unknown
**NCT02064491**	Phase 2	Randomized, open label	EGFR-TKI Chemotherapy	Erlotinib, Cisplatin, Carboplatin, Docetaxel, Paclitaxel, Pemetrexed	NSCLC	18	PFS	Completed
**NCT04923906**	Phase 3	Randomized, open label	EGFR-TKI Chemotherapy	Aumolertinib Placebo	NSCLC	624	PFS^a^	Not yet recruiting
**NCT03050411**	Phase 1	Single-arm, open label	EGFR-TKI VEGFR2 inhibitor	Apatinib, Erlotinib, Gefitinib, Osimertinib	NSCLC	30	Optimal dosage, PFS	Unknown
**NCT04358562**	Phase 2	Randomized, open label	EGFR-TKI VEGFR inhibitor	Gefitinib, Anlotinib	*EGFR* mutation-positive NSCLC	240	PFS	Not yet recruiting
**NCT04197076**	N/A	Cohort	EGFR-TKI Immunotherapy	Anti-PD-1 or anti-PD-L1 EGFR-TKI, ALK inhibitor, ROS1 inhibitor	NSCLC	200	DFS, pCR rate	Unknown
**NCT02630186**	Phase 1 Phase 2	Single-arm, open label	EGFR-TKI Immunotherapy	Rociletinib, MPDL3280A	NSCLC	3	Treatment-emergent adverse events, Cmax, Tmax, Cmin	Terminated

a PFS as assessed by Independent Review Committee.

Cmax, maximum concentration; Cmin, minimum concentration; DFS, disease-free survival; EGFR, epidermal growth factor receptor; NSCLC, Non-small Cell Lung Cancer; pCR, pathologic complete response; PFS, progression-free survival; Tmax, time to maximum concentration; TKI, tyrosine kinase inhibitors; VEGFR, vascular endothelial growth factor receptor.

### Combined application of EGFR-TKI and anti-VEGF drugs

A couple of anti-VEGF drugs are being explored in combination with EGFR-TKIs in early phase clinical trials. A phase I study NCT03050411 aims to evaluate the dosage and efficacy of apatinib, a TKI that selectively inhibits VEGFR-2, when given in combination with EGFR-TKI in the treatment of EGFR-TKI-resistant advanced non-squamous non-small cell lung cancer. A phase II clinical trial NCT04358562 aims to evaluate the efficacy and safety of gefitinib combined with anlotinib, a domestic oral small molecule inhibitor of multi-receptor tyrosine kinase, versus gefitinib alone in advanced non-squamous NSCLC patients whose *EGFR* mutation was not cleared in plasma ctDNA after 8 weeks of gefitinib first-line treatment, to provide a clinical basis for a new and tolerable treatment that can prolong survival of patients with advanced NSCLC (**Table 1**).

### EGFR-TKI combined immunotherapy

To prolong the response duration of *EGFR* mutant NSCLC and delay the onset of drug resistance, clinical trials combining EGFR-TKIs and immunotherapy were conducted. As part of the CHECKMATE 012 study, 21 patients with *EGFR* mutation-positive NSCLC (20 patients who received erlotinib pretreatment and 1 patient who did not receive EGFR-TKI treatment) were evaluated for safety and efficacy (68). 3 mg/kg nivolumab and 150 mg erlotinib were given every 2 weeks until disease progression or unacceptable toxicity. Common adverse events include skin rash, fatigue, paronychia, diarrhea and skin cracks. It has been reported that 19% of patients experienced grade 3 toxicity, with no grade 4 toxicity. ORR at 24 weeks was 19%, PFS was 51%, and OS at 18 months was 64%. In addition, of the 20 patients pretreated with EGFR-TKI, 3 patients achieved partial remission, with a median remission time of 60.1 weeks. The treatment time of the 2 patients with T790M-negative repeat biopsy was 61 weeks and 108 weeks, respectively. One patient who did not receive EGFR-TKI obtained partial remission and continued treatment for 72.3 weeks. Among patients pretreated with EGFR-TKI, ORR was 67% in T790M-positive patients and 21% in T790M-negative patients. The ORR of EGFR-TKI-naive patients was 70%. However, this combination is associated with a high incidence of interstitial lung disease (ILD), with a prevalence of 38%. Among the 13 patients with ILD, 5 were grade 3/4, and there were no fatal cases. Most of the cases were treated with high-dose corticosteroids and drug withdrawal. The median time to onset of ILD was 69 days. Considering that the incidence of ILD associated with osimertinib is about 2%-3%, the incidence associated with nivolumab monotherapy is less than 2%, and the incidence remains high. In terms of race, although the higher incidence rate associated with EGFR-TKIs has been considered to be related to Asian ethnicities, especially Japanese patients, the underlying mechanism is still under investigation.

Durvalumab is another immunotherapy drug that is being explored in combination with EGFR-TKI. In an ongoing phase 1b dose-escalation and dose-expansion study, safety data support the combined use of 10 mg/kg durvalumab and 250 mg gefitinib (69). During the dose-expansion phase, 10 patients in group 1 who did not receive EGFR-TKI were treated with durvalumab (10 mg/kg every 2 weeks) plus gefitinib (250 mg once a day), or gefitinib was used as a single agent in the two groups for 4 weeks initially, and then durvalumab plus gefitinib was used at the same time. This combination is tolerable in most cases, and more common adverse events of any grade were observed in 80% and 60% of treatment-related adverse events in each group. However, grade 3/4 adverse events were elevated alanine aminotransferase (ALT) in the two groups (70% in group 1 and 60% in group 2) and elevated aspartate aminotransferase (AST, group 1 40%, group 2 is 50%). The increase in ALT/AST is controlled by dose interruption and corticosteroid use. In terms of efficacy, the ORR of group 1 and group 2 were 77.8% and 80.0%, respectively.

The combination of EGFR-TKI and immunotherapy is also being explored in NSCLC. An ongoing observational clinical study (NCT04197076) is investigating the safety and efficacy of gefitinib as neoadjuvant therapy in patients with stage IIIA NSCLC. A phase 1b/2 trial (NCT02630186) aims to evaluate the safety and efficacy of rociletinib administered in combination with MPDL3280A.The primary purpose of the Phase 1 portion of the study is to observe the safety of the combination of rociletinib and MPDL3280A in patients with *EGFR* mutation-positive NSCLC. The primary objectives of the Phase 2 portion of the study are to evaluate the safety and anti-tumor effects of the combination of rociletinib and MPDL3280A, at the best doses for the combination determined in Phase I, in patients with EGFR mutation-positive NSCLC.

## Other novel therapies

In addition to the EGFR-TKI combination therapies discussed above, alternative treatment strategies, such as nanomedicine, are emerging as potential promising therapies for lung cancer. Biodegradable nanoparticles (<1 μm) with anticancer drugs encapsulated represent a novel approach which enables effective targeted drug delivery without excess toxicities to normal cells. Anticancer drugs are either adsorbed to the surface of the nanoparticle, or encapsulated in a polymer-based cavity, and delivered to the targeted tissue either through intratumoral injection or inhaler (73). In an *in vivo* study, a thermosensitive nanoparticle delivery system with cisplatin and paclitaxel loaded has demonstrated good efficacy with acceptable safety as *in situ* treatment in a lung cancer mouse xenograft model, which suggests the potential of combining nanoparticle technology with conventional chemotherapy in the treatment of lung cancer (74).

In addition to delivery of conventional anti-cancer drugs, nanoparticles can also be used to deliver non-coding RNAs (ncRNAs) to tackle EGFR-TKI resistance. Dysregulated ncRNA have been shown to play a regulatory role in EGFR‐TKI resistance in lung cancer through modulating multiple signaling pathways, including the PI3K/AKT/mTOR, JAK/STAT, Ras/Raf/MEK/ERK signaling pathways. Therefore, ncRNAs have been explored as a potential therapeutic target for overcoming EGFR-TKI-resistance in lung cancer (75). In an *in vitro* study, nanoparticles encapsulating doxorubicin and short-interfering RNA were able to silence multi-drug resistance genes in lung cancer, highlighting the potential of combining nanomedicine with ncRNA in overcoming resistance to targeted therapy (76). Another study using nanoparticles to deliver short-interfering RNA against an established oncogenic long noncoding RNA (differentiation antagonizing noncoding RNA) achieved over 90% silencing, which resulted in reduced cell migration and invasion, and improved EGFR inhibitor response (77). Although still in early stage, these promising results shed light on the potential of RNA interference through nanoparticle delivery system as a novel approach for lung cancer treatment.

## Predictive biomarkers for EGFR-TKI sensitivity

Although EGFR-TKI combination treatment for patients with advanced NSCLC with EGFR-sensitive mutations is extensively studied, so far, no accurate and effective prognostic biomarker has been established in this setting, which represents an unmet clinical need. In addition to exploring prognostic new biomarkers, studies suggest that inflammation, immune response, and tumor immune microenvironment are associated with the prognosis of patients with various malignancies (55, 78–81). Increased research is devoted to finding biomarkers for the prognosis of patients with NSCLC, and multiple markers have been discovered to predict disease progression status and patient prognosis from different perspectives. These include serum biomarkers C-reactive protein (CRP), inflammatory markers neutrophil to lymphocyte ratio (NLR) and lymphocyte to monocyte ratio (LMR), and nutritional level markers prognostic nutritional index (PNI), etc.

The level of CRP secreted by the liver increases rapidly in response to trauma, inflammation, and infection, and also decreases rapidly as the condition subsides. A retrospective study on patients with EGFR-sensitive mutant advanced NSCLC treated with EGFR-TKI found that, compared with patients with low pre-treatment serum CRP levels, patients with high pre-treatment serum CRP levels had a significantly poorer response to EGFR-TKI (65, 81).The results suggest that pre-treatment serum CRP levels can independently predict the prognosis of patients with advanced NSCLC who are treated with EGFR-TKIs.

The inflammatory index marker NLR can be used to reflect the body’s inflammation. When the peripheral blood neutrophil count is high or the lymphocyte count is low, the ratio becomes unbalanced, indicating a high inflammatory response. High levels of neutrophils will affect the anti-tumor effects of lymphocytes, while low lymphocyte level is not sufficient for the immune system to clear tumor cells. The above two factors can promote tumor growth. Gui Nan and Fausto Meriggi found lower NLR levels are associated with significantly longer PFS and OS in patients with NSCLC who received EGFR-TKI (82, 83). Therefore, NLR level prior to initial treatment has been explored as an independent factor of survival and prognosis in patients with NSCLC who received EGFR-TKI. The LMR is simple measurement and objective indicator of inflammation, and is an established independent prognostic factor in patients with multiple malignancies (84). A retrospective study found that high LMR levels are significantly related to the better prognosis of patients with advanced *EGFR* mutation-positive NSCLC treated with EGFR-TKI (78, 79). Therefore, LMR can be used to as a prognostic indicator for EGFR-TKI treatment, but the specific cut-off value for stratifying high or low LMR needs to be determined by further research.

PNI is an index that can comprehensively reflect the inflammation and nutritional status of patients with cancer (80). It is reported that PNI is related to tumor progression hence is of prognostic value in a variety of malignant tumors such as digestive tract tumors, pancreatic cancer and lung cancer (84–89). Research by Jin and colleagues found that the lower PNI is associated with poor prognosis in patients with EGFR-sensitive mutant NSCLC who received EGFR-TKI (90). Therefore, it might be worth applying the PNI prior to initiating EGFR-TKI treatment as routine clinical practice.

In addition, new biomarkers are needed to differentiate patients with *EGFR* mutation-positive lung cancer who are resistant to EGFR-TKIs. Although patients with *EGFR* mutation-positive NSCLC are commonly sensitive to EGFR-TKIs initially, the development of acquired resistance is inevitable (38). The mechanisms of acquired resistance to EGFR-TKIs can be classified into point mutations in target genes, activation of alternative signaling pathways, and phenotypic or histological transformation. T790M is a point mutation at position 790 on exon 20 of the EGFR kinase domain, where threonine is replaced by methionine, which subsequently activates downstream signaling pathways (91, 92). Residue 790 that controls entry of EGFR-TKIs into the ATP-binding pocket will increase their affinity for ATP and cause acquired resistance to targeted drugs when turned off. Detection of drug-resistant mutations by gene profile screening and by comprehensive evaluation based on system examination could potentially provide improvement to the efficacy of EGFR-TKIs in patients with NSCLC.

## Conclusion

By now, most EGFR-TKIs aim to target the biological functions of EGFR caused by kinase activation, and to induce cytotoxicity of cancer cells, yet drug-resistant mutations, together with changes in the stability of EGFR still limits the use and efficacy of EGFR-TKIs in clinical practice. The first-generation of EFGR-TKI such as gefitinib proved effective in the most common 19del and L858R mutations, but not in resistant mutations such as T790M, which accounts for 60% of drug-resistant *EGFR* mutations (93). New EGFR-TKIs represented by osimertinib and afatinib are promising solutions to the resistant mutations. Moreover, monoclonal antibodies targeting EGFR and its downstream signaling pathways were reported in treating colorectal cancer, head and neck carcinomas, but few reports comprehensively investigated the clinical outcomes in NSCLC (94). This review discussed EGFR-TKI resistance mechanisms and its clinical implications in NSCLC, summarized current EGFR-TKI-based combination therapy regimens and overviewed novel treatment approaches including nanoparticles encapsulating non-coding RNAs for overcoming EGFR-TKI resistance in NSCLC. In summary, EGFR-TKIs combination therapies are potent treatment strategies against NSCLC, and further research are needed to overcome resistance and other barriers that hinder their clinical application.

## Author contributions

WP, GZ, and YF provided the direction and guidance of this manuscript. CY and QP wrote the whole manuscript. JY, BS, and ZZ made significant revisions to the manuscript. All authors contributed to the article and approved the submitted version.
